# Value Realization of Grassland Ecosystem Products in the Karst Desertification Control Area: Spatial Variability, Drivers, and Decision‐Making

**DOI:** 10.1002/ece3.71168

**Published:** 2025-03-27

**Authors:** Yongyao Li, Anjun Lan, Kangning Xiong, Wenfang Zhang, Shuai Xiang, Baoshan Zhang

**Affiliations:** ^1^ Schools of Karst Science and Geography/State Engineering Technology Institute for Karst Desertfication Control Guizhou Normal University Guiyang China; ^2^ Bijie Institute of Science and Technology Information Bijie Science and Technology Bureau Bijie China

**Keywords:** grassland, InVEST model, karst desertification control, random forest model, value realization of ecosystem products

## Abstract

Transforming the ecological advantages of grassland ecosystems into economic benefits while ensuring their long‐term health is an urgent but challenging question, particularly in karst areas characterized by significant spatial heterogeneity. This study selected three representative karst desertification control (KDC) areas within the South China Karst (SCK) as the research focus. Utilizing the quantified values of ecosystem products and their realization rates, we applied a random forest model to analyze the influencing factors. We found that: (1) The gross ecosystem products (GEP) of grassland per unit area increase with the severity of karst desertification. Conversely, the value realization rate decreases as the grade of karst desertification increases, contradicting the theoretical assumption that higher GEP correlates with a high value realization rate. (2) Water, soil, climate, and bare rock coupled with human activities (e.g., ecological engineering) affect the structure of the grassland GEP, which, in turn, affects the value realization rate of grassland ecosystem products in the KDC area. Based on our findings, we suggest that economic leapfrogging can be achieved through artificial grassland engineering in ecologically disadvantaged areas, challenging the conventional belief that a more fragile ecological environment results in poorer economic advantages. However, it is important to note that plant species diversity in artificial grassland in areas with severe karst desertification is low, and the trade‐off and equity between ecology and economy must be carefully considered in future planning. Our findings can serve as a reference for subsequent phases of grassland ecosystem restoration for sustainability in ecologically fragile areas, particularly within the KDC regions.

## Graphical abstract

The gross ecosystem product of grassland decreased with the increasing karst desertification grade. The market contribution of grassland gross ecosystem product declined with increasing karst desertification levels; The rock exposure rate has little effect; instead, climate and industrial models have a great driving effect.

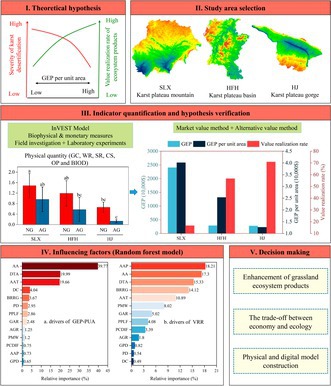

## 1 Introduction

Grasslands, which cover approximately 40% of the global terrestrial ice‐free surface, not only provide food for humans through grazing but also contribute significantly to biodiversity and offer numerous valuable ecosystem services Bai and Cotrufo 2022. Globally, about one billion people rely on grassland resources for their livelihoods (Buisson et al. 2022). However, the vulnerability of grassland ecosystems, compounded by irrational human economic activities, has led to their degradation (Schils et al. 2020), thereby threatening human well‐being (Bardgett et al. 2021). The value realization of ecosystem products seeks to transform the burden of environmental protection into opportunities for economic development, thereby achieving both ecological and economic sustainability (Zhang et al. 2024). In the context of the United Nations Decade on Ecosystem Restoration (2021–2030), recognizing grassland ecosystems as core value creators and integrating them into the modern ecological economic system of production, distribution, exchange, and consumption is crucial for benefiting both people and nature in the pursuit of sustainability.

Karst grasslands constitute a significant component of the global grassland ecosystem. The ecological and economic functions of grasslands are indispensable for addressing ecological degradation in karst regions, such as karst desertification (Lan et al. 2001). Over the past century in the stony semidesert region of the Dinaric karst, farmers have transformed extensive pastures into meadows to restore the ecological environment and improve agricultural conditions (Gams and Matej 1999). In the Magnesian region of England, local governments achieved cost‐effectiveness and vegetation sustainability by establishing permanent low‐maintenance grasslands for the restoration of limestone quarrying (Richardson and Evans 1986). In the early 21st century, the Chinese government implemented ambitious ecological projects—including converting farmland back to grassland, establishing new grasslands, and developing herbivorous animal husbandry—in the South China Karst (SCK) region to promote greening, optimize industrial structure, and enhance farmers' livelihoods (Qiu et al. 2021). Maintaining grassland ecosystem health and maximizing ecosystem products for human well‐being in the KDC area represent an urgent and significant challenge, particularly in light of current and future ecosystem restoration imperatives. Achieving this goal necessitates a diverse range of theories, methods, and pathways for the value realization of ecosystem products.

In recent years, there has been growing global attention on achieving dual ecological and economic sustainability through the value realization of grassland ecosystem products. Primarily, the cognition and quantification of grassland ecosystem products have garnered widespread attention. Scholars generally recognized the importance of grasslands for food supply, carbon sequestration, biodiversity, and pollination, and other ecosystem services (Bengtsson et al. 2019; Zhao et al. 2020). Based on these findings, researchers often estimate the physical quantity of grassland ecosystem products using remote sensing monitoring (Masenyama et al. 2022; Oberrneier et al. 2019), sample plot monitoring (Klein et al. 2020; Prangel et al. 2023), and model simulations (Paruelo et al. 2024). The development of remote sensing technology, in particular, has provided significant technical support for the accurate monitoring of grassland ecosystem products. For example, Sentinel‐2 has been widely used to capture grassland phenological characteristics due to its temporal and spatial resolution and cost‐free availability (Bartold and Kluczek 2023), which has improved data accuracy while reducing labor and material costs. Along with the development and application of models such as machine learning, the “Remote Sensing + Deep Learning” predictive paradigm has been established (Muro et al. 2022). As research progresses, there has been a gradual shift from biophysical valuation, which emphasizes ecological attributes and their intrinsic value, to valuations focusing on economic attributes and their utility. For example, Huber et al. (2022) emphasized the importance of accounting for the economic value of grassland ecosystem products in decision‐making for grassland management and incentives. Schirpke et al. (2020) assessed the value of grassland ecosystem products in the Autonomous Province of South Tyrol, Italy, to predict the economic loss resulting from the transition of grassland to woodland. Direct measurement of grassland ecosystem products in monetary terms provides objective scientific evidence, countering subjective cognitive biases.

In addition to supply capacity, researchers universally analyze the factors influencing grassland ecosystem products from both natural and anthropogenic perspectives. There is a consensus among researchers that the components, structures, and processes of grassland ecosystems under varying site conditions influence their functions. For example, increased biodiversity directly enhances carbon sequestration in grasslands (Hungate et al. 2017), and elevated soil microbial composition positively affects nutrient cycling and plant growth (Pommier et al. 2018). Conversely, scholars have increasingly focused on the crucial role of essential biodiversity variables (EBVs) and essential climate variables (ECVs) in monitoring and understanding changes in grassland ecosystem dynamics (Dabrowska‐Zielinska et al. 2017; Pereira et al. 2013). Regarding human factors, land intensification has been found to enhance grass production in temperate and subtropical rangelands while simultaneously reducing grassland conditioning and support services (Neyret et al. 2021). This case also confirms that land intensification reduces the multifunctionality of permanent grasslands (Schils et al. 2022). Still, grassland management plays a crucial role in ecosystem product provision and the enhancement of ecological diversity. For example, field monitoring has shown that grazing, fertilization, and mowing significantly affect grassland biological production, soil quality, and both animal and plant diversity (Vooren et al. 2018). Additionally, stakeholder perceptions and views lead to trade‐offs in the provision of grassland ecosystem products (Schmitt et al. 2021).

**FIGURE 1:**
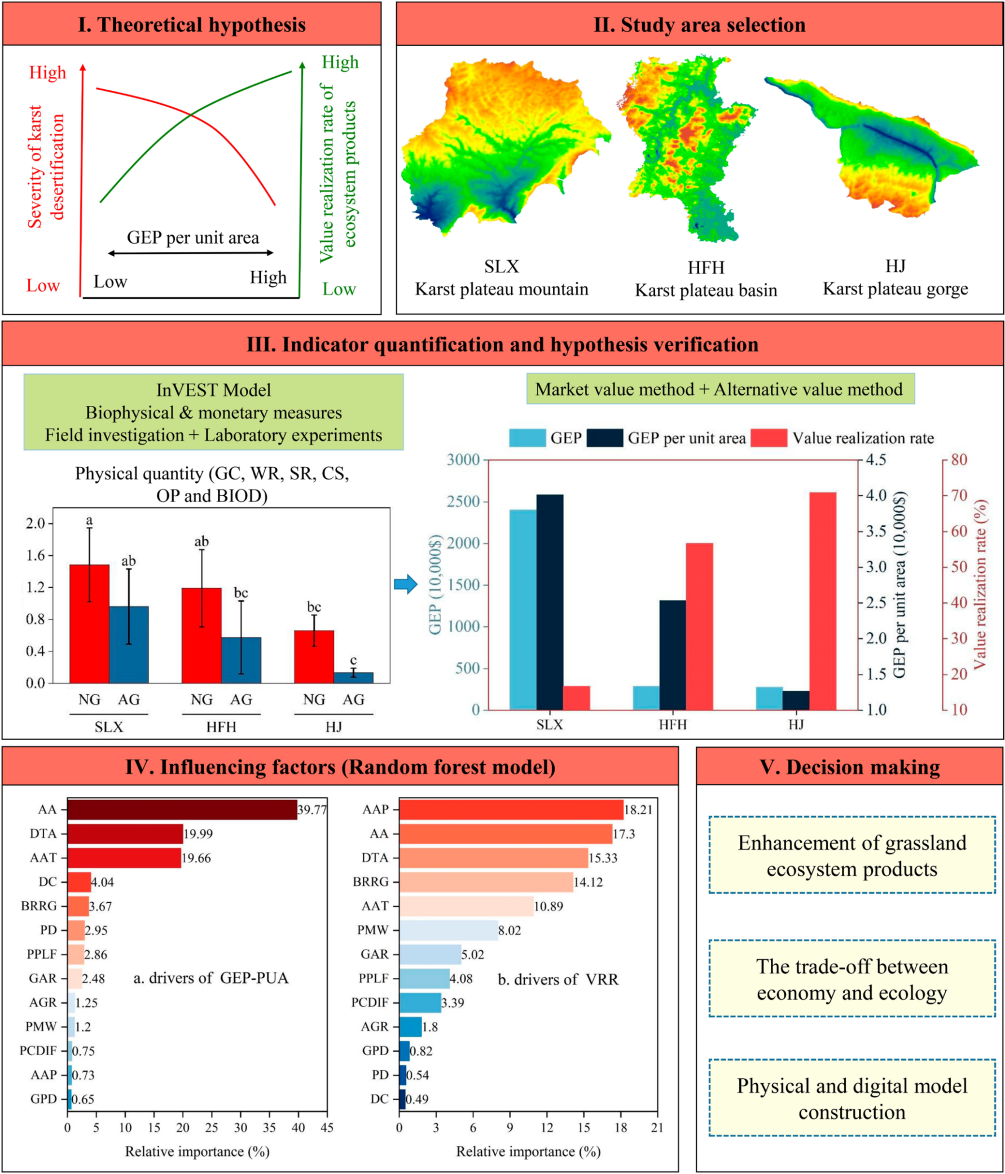
Research framework and technical approach. AA, Average altitude; AAP, Average annual precipitation; AAT, Average annual temperature; AG, Artificial grassland; AGR, Artificial grassland ratio; BIOD, Biodiversity; BRRG, Bare rocky ratio of grassland; CS, Carbon sequestration; DC, Distance to county; DTA, Distance to tourist attractions; GAR, Grassland area ratio; GC, Grazing capacity; GEP‐PUA, Gross ecosystem products per unit area; GPD, Grassland patch density; HFH, Hongfenghu; HJ, Huajiang; NG, Natural grassland; OP, Oxygen production; PCDIF, Per capita disposable income of farmers; PD, Population density; PMW, Proportion of migrant workers; PPLF, Proportion of pastoralists in the labor force; SLX, Salaxi; SR, Soil retention; VRR, Value realization rate; WR, Water retention.

Despite considerable prior research, scholars have predominantly focused on the physical volume and enhancement mechanisms of grassland ecosystem products, with limited attention given to their economic value for both people and nature. Notably, a systematic understanding of the market conversion rate and enhancement strategies for these products remains lacking, particularly in the karst region, where spatial heterogeneity is highly pronounced. To fill this knowledge gap, we selected three demonstration areas within the SCK region for the study. We initially propose the theoretical assumption that a greater resource endowment in grasslands correlates with an increased capacity for ecosystem product supply and a higher rate of value realization for these products. Based on this theoretical assumption, we employed bivariate correlation analysis and a random forest model to analyze the influencing factors by quantifying the value of grassland ecosystem products and their respective value realization rates. The objectives of this research are twofold: (1) to validate our theoretical hypotheses through case studies, and (2) to analyze the influencing factors affecting grassland ecosystem products and their value realization rates, ultimately proposing countermeasures. The technical approach is illustrated in Figure 1.

## 2 Material and Methods

### 2.1 Study Area

**FIGURE 2:**
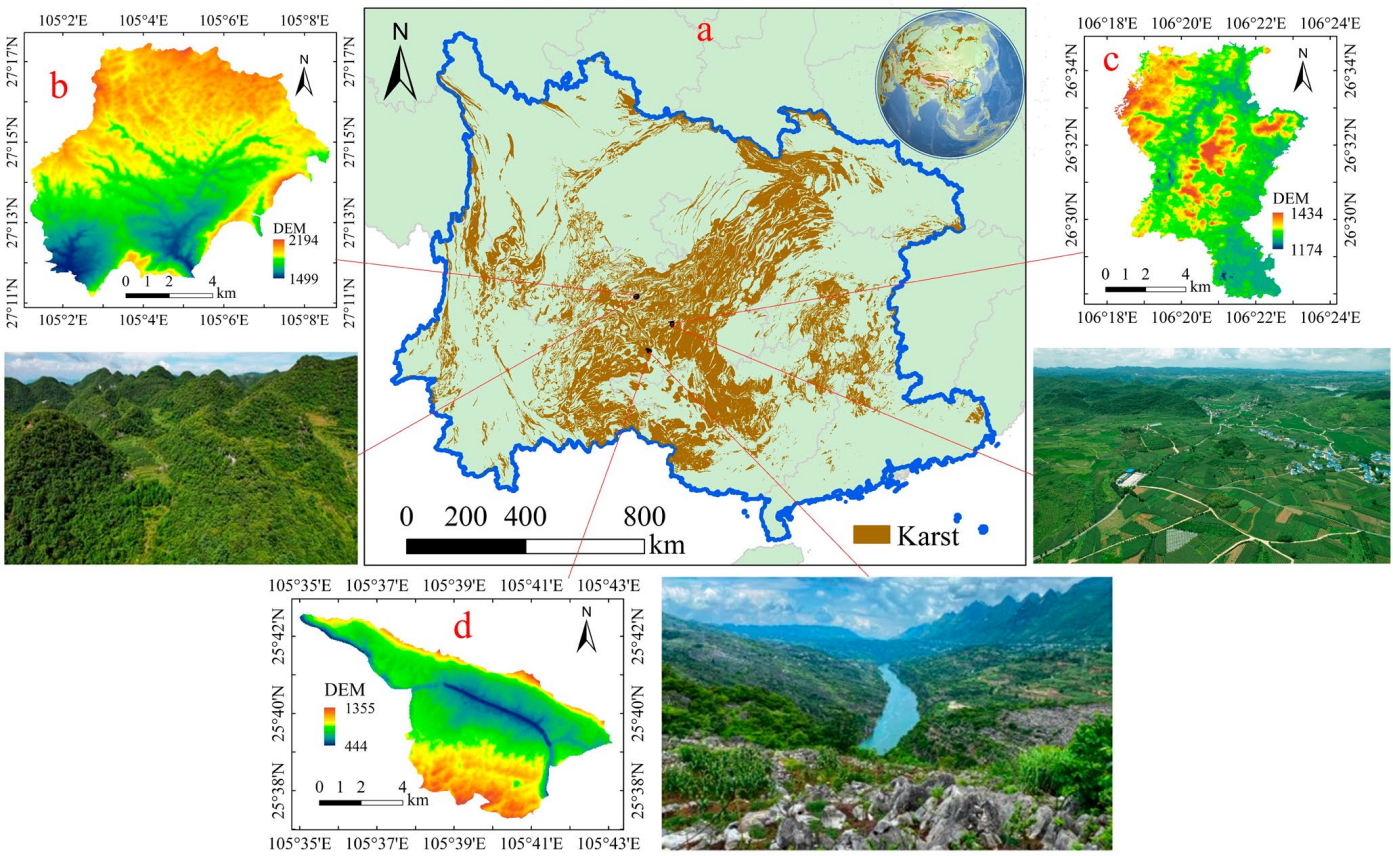
Study area. (a) refers to the location of SCK in the world; (b–d) represent the location, DEM, and landform of Salaxi, Hongfenghu, and Huajiang study areas, respectively. Photo by Qi Wang.

The study areas of Salaxi, Hongfeng Lake, and Huajiang are situated in the core region of the SCK, centered on the Yunnan–Guizhou Plateau (Figure 2a). This region is distinguished by extensive carbonate rock formations, diverse geomorphic features, and considerable spatial heterogeneity. It serves as a critical ecological barrier in the upper reaches of both the Yangtze and Pearl Rivers. Furthermore, the region experiences a humid subtropical monsoon climate and is home to a large population. Detailed information about the three study areas is provided in Table 1.

**TABLE 1:** The detailed information of the three study areas.

Information	Salaxi	Hongfenghu	Huajiang
Landform	Karst plateau mountain (Figure 2b)	Karst plateau basin (Figure 2c)	Karst plateau gorge (Figure 2d)
Area	86.27 km^2^	60.44 km^2^	51.62 km^2^
Proportion of karst area	73.94%	94.59%	87.92%
Average elevation	1878 m	1300 m	947 m
Average annual temperature	12.8°C	15.5°C	18.4°C
Average annual precipitation	984.4 mm	1,192.5 mm	1,100 mm
Karst desertification level	Potential‐light	Light‐moderate	Medium‐severe
Population (2023)	31,597	22,439	7361
Natural grassland area (2023)	593.11 hm^2^	95.14 hm^2^	209.27 hm^2^
Artificial grassland area (2023)	4.89 hm^2^	17.02 hm^2^	22.09 hm^2^
Timing of the establishment of the study area and implementation of the KDC project	2005	2000	1996

### 2.2 Research Methods

#### 2.2.1 Accounting for GEP and the Value Realization Rate of Ecosystem Products

The primary value of grasslands in the KDC area lies in their ability to provide an ecological safety shield for local economic and social development, enhance biodiversity, and support the development of herbivorous livestock. Consequently, this study considers various aspects of grassland value, including grazing capacity, water retention, soil retention, carbon sequestration, oxygen production, biodiversity, and landscape tourism, to reflect the grassland ecosystem products.

#### 2.2.2 Grazing Capacity Value

This analysis is based on field statistics to quantify physical quantities and employs the market value method to assess value quantities. The calculation formula is presented as follows:

(1) $$\text{Physical quantity}\;\;\mathrm{GC}={\mathrm{GC}}_{\text{NGPUA}}\times A_{\mathrm{NG}}+{\mathrm{GC}}_{\text{AGPUA}}\times A_{\mathrm{AG}}$$

(2) $$\text{Value}\;\;V_{\mathrm{gc}}=\mathrm{GC}\times V$$

where GC denotes the grazing capacity (cattle), ${\mathrm{GC}}_{\text{NGPUA}}$ and ${\mathrm{GC}}_{\text{AGPUA}}$ are the grazing capacity of natural grassland and artificial grassland per unit area (Cattle/hm^2^), respectively, $A_{\mathrm{NG}}$ and $A_{\mathrm{AG}}$ represent the area of natural grassland and artificial grassland (hm^2^), respectively. $\;V_{\mathrm{GC}}$ is the total value of the grazing capacity ($). V refers to the unit price of cattle, which is 2043.51$/cattle based on the average exchange rate of RMB and USD in 2023 (7.0467CNY equals $1, the same below), combined with the average price of local cattle in the study area.

#### 2.2.3 Water Retention Value

Based on the accounting method for water yield in karst areas proposed by Wang et al. (2020), the physical quantity was quantified using the InVEST model (https://naturalcapitalproject.stanford.edu/invest/). The market value method was employed to assess value quantities. The calculation formula is presented as follows:

(3) $$\begin{array}{c} \text{Physical quantity}\;\;\mathrm{WR}=\min\left(1,\frac{249}{\text{Velocity}}\right)\\ \times \min\left(1,\frac{0.9\times \mathrm{TI}}{3}\right)\times \min\left(1,\frac{\text{Ksat}}{300}\right)\times Y_{x} \end{array}$$

(4) $$\mathrm{TI}=\log\left(\frac{\text{DrainageArea}}{\text{SiolDepth}\times \text{Percent}\_\text{slope}}\right)$$

(5) $$Y_{x}=\left(1-\frac{{\mathrm{AET}}_{x}}{P_{x}}\right)\times P_{x}$$

(6) $$\text{Value}\;V_{\mathrm{wr}}=\mathrm{WR}\times P_{w}$$

where *WR* (t) represents the water retention, *Velocity* indicates the flow coefficient, *TI* is the topographic index, *Ksat* denotes the saturated hydraulic conductivity of the soil, $Y_{x}$ describes the water yield of patch x (mm·yr.^−1^), DrainageArea is the catchment area of patch x (hm^2^), SiolDepth is the soil depth (cm), and Percent_slope is the percentage of slope (%). ${\mathrm{AET}}_{x}$ measures the actual annual evapotranspiration of patch x (mm·yr.^−1^), and $P_{x}$ is the annual precipitation of patch x (mm·yr.^−1^). $V_{\mathrm{wr}}$ refers to the water retention value. $P_{w}$ is the price of water, with reference to the price of water in the study area in 2023, it is taken as $0.43/t.

#### 2.2.4 Soil Retention Value

Drawing on the method established by Feng et al. (2016) for accounting soil retention in karst areas and utilizing the InVEST model, the Revised Universal Soil Loss Equation (RUSLE) was employed to quantify physical volume, while the shadow engineering method and alternative value method were utilized to assess value volume. The calculation method is presented as follows:

(7) $$\text{Physical quantity}\;\;Q_{\mathrm{sr}}=R\times K\times \mathrm{LS}\times \left(1-C\times P\right)$$

(8) $$V_{\mathrm{sr}}=V_{1}+V_{2}$$

(9) $$V_{1}=\left(\frac{Q_{\mathrm{sr}}}{\rho }\right)\times r\times \beta_{1}$$

(10) $$V_{2}=\sum \left(Q_{\mathrm{oi}}/r_{i}\right)\times \beta_{2}$$

(11) $$\text{Value}\;Q_{\mathrm{oi}}=Q_{\mathrm{sr}}\times C_{i}$$

where $Q_{\mathrm{sr}}$ (t·yr.^−1^) refers to the amount of soil retention. *R*, *K*, *LS*, *C*, and *P* represent rainfall erosive force, soil erosion coefficient, topographic coefficient, vegetation cover and management factor, and soil and water conservation measure factor, respectively. $V_{\mathrm{sr}}$ ($) refers to the total value of soil retention, $V_{1}$ ($) describes the value of sediment reduction, and $V_{2}$ ($) represents the value of fertilizer retention. ρ (t/m^3^) is the soil bulk weight, which is 1.4, 1.45, and 1.21 in the Salaxi, Hongfenghu, and Huajiang regions, respectively, based on the results of laboratory experiment. r (%) is the percentage of sediment lost by soil erosion and deposited in reservoirs, rivers, and lakes, refer to Li et al. (2023), take 0.24. $\beta_{1}$ is the project cost per unit of reservoir capacity, which is taken as 5.96$/t; $Q_{\mathrm{oi}}$ is the annual amount of fertilizer preservation (t·yr.^−1^). $r_{i}$ is the N, P, K, and organic matter content in fertilizer and organic matter, which is taken as 14%, 15%, 50%, and 100% according to “The specifications for assessment of grassland ecosystem services” (NAMR 2021. $\beta_{2}$ indicates the prices of fertilizer and organic matter, according to the survey in the study area, the price of diammonium phosphate is $434.25/t, the price of potassium chloride fertilizer is $333.49/t, and the price of organic matter is $121.33/t. $C_{i}$ (%) is the N, P, K, and organic matter content of the soil, according to the laboratory experiments, the content of N in Salaxi, Hongfenghu, and Huajiang study was 0.2%, 0.22%, and 0.35%, respectively; the content of P was 0.031%, 0.0321%, and 0.0325%, respectively; the content of K was 1.09%, 1.17%, and 1.29%, respectively; and the content of organic matter was 40.95%, 41.82%, and 42.65%, respectively.

#### 2.2.5 Carbon Sequestration Value

The physical and value quantities of carbon sequestration encompass four components: above‐ground vegetation, below‐ground vegetation, soil, and rock. The formula is as follows:

(12) $$\text{Physical quantity}\;Q_{T_{{\mathrm{co}}_{2}}}=Q_{c1}+Q_{c2}+Q_{c3}+Q_{cckarst}$$

(13) $$Q_{c1}=0.45\times A_{g}\times G_{e1}$$

(14) $$Q_{c2}=0.45\times A_{g}\times G_{e2}$$

(15) $$Q_{c3}=S_{d}\times A_{g}÷100$$

(16) $$Q_{\text{ckarst}}=0.2198\times A_{g}$$

(17) $$\text{Value}\;V_{{\mathrm{co}}_{2}}=Q_{T_{{\mathrm{co}}_{2}}}\times P_{{\mathrm{co}}_{2}}$$

$Q_{T_{{\mathrm{co}}_{2}}}$ (t) is the amount of carbon sequestered in grassland. $Q_{c1}$, $Q_{c2}$, $Q_{c3}$, and $Q_{\text{ckarst}}$ (t) are the amount of carbon sequestered by above‐ground vegetation, below‐ground vegetation, soil carbon sequestration, and karst carbon sequestration, respectively. 0.45 = 1.63 × 0.2727, which is the international common index conversion method, interpreted as 1.63 g of CO_2_ fixed in 1 g of dry matter, and 0.2727 as 27.27% carbon in the fixed CO_2_. $A_{g}$ (hm^2^) denotes the grassland area. $G_{e1}$ and $G_{e2}$ (t/hm^2^) measures the dry matter quantity of aboveground and below‐ground of grassland, respectively. $S_{d}$ (g/m^2^) is the soil organic carbon density. 0.2198 (t/hm^2^/a) represents the rate of carbon sequestration in karst areas. $V_{{\mathrm{co}}_{2}}$ ($) quantifies the value of carbon sequestration in grassland. $P_{{\mathrm{co}}_{2}}$ indicates the carbon trading market price, refer to Li et al. (2023), take $4.77/t.

#### 2.2.6 Oxygen Production Value

According to the chemical equation of photosynthesis, it is established that for each mole of CO_2_ absorbed by a plant, 1 mol of O_2_ is released. The formula is as follows:

(18) $$\text{Physical quantity}\;Q_{\mathrm{op}}=M_{O_{2}}/M_{{\mathrm{CO}}_{2}}\times \left(Q_{c1}+Q_{c2}\right)$$

(19) $$\text{Value}\;V_{\mathrm{op}}=Q_{\mathrm{op}}\times C_{O}$$

In this equation, $Q_{\mathrm{op}}$ (t) refers to the amount of oxygen released from grassland. $M_{O_{2}}/M_{{\mathrm{CO}}_{2}}$ = 32/44, which is the coefficient of conversion of *CO*_*2*_ to *O*_*2*_, $Q_{c1}$ and $Q_{c2}$ (t) represent the amount of carbon sequestration from aboveground vegetation and underground vegetation, respectively. $V_{\mathrm{op}}$ ($·yr.^−1^) indicates the oxygen production value. $Q_{\mathrm{op}}$ (t·O_2_·yr.^−1^) is the amount of oxygen released. $C_{O}$ describes the industrial oxygen price ($/t), according to Li et al. (2023), we take $207.52/t.

#### 2.2.7 Biodiversity Value

Plant diversity is accounted for here due to limitations in data sources. The Shannon–Weiner diversity index was used for measurement. The formula is as follows:

(20) $$\text{Physical quantity}\;\;{\mathrm{GDI}}_{\mathrm{isw}}=-\sum_{i}^{S}\left(n_{i}÷N\right)\ln\left(n_{i}÷N\right)$$

(21) $$\text{Value}\;V_{\mathrm{bio}}=\sum A_{i}\times S_{\mathrm{isw}}$$

Where, ${\mathrm{GDI}}_{\mathrm{isw}}$ represents the Shannon–Weiner diversity index of pixel *I*; $n_{i}$ denotes the number of individuals of species *I*; *N* indicates the sum of the number of individuals of all species; *S* is the number of species. $V_{\mathrm{bio}}$ ($·yr.^−1^) refers to the value of grassland biodiversity, $S_{\mathrm{iSW}}$ ($·yr.^−1^) is the opportunity cost of annual species loss per unit area of grassland type *i*, assigned a value in terms of the rank of the Shannon–Weiner diversity index. The price corresponding to the Shannon–Weiner diversity index is derived from “The specifications for assessment of grassland ecosystem services”. $A_{i}$ (hm^2^) is the area of the *i*‐th type of grassland.

#### 2.2.8 Landscape and Tourism Value

The cultural ecosystem service value of grasslands in the KDC area was assessed using the alternative market value method due to the challenges associated with direct assessment. Drawing on the work of Hirons et al. (2016) regarding the perception of cultural ecosystem services, consumer expenditure derived from the travel cost method was utilized as a measure. Given that the study area encompasses a complete ecosystem, including grasslands, forests, farmlands, and other ecosystems, the value of cultural services is extracted here based on the proportion of land area. The formula is presented as follows:

(22) $$\text{Value}\;V_{\mathrm{glt}}=\frac{A_{g}}{A}∙V_{\mathrm{lt}}$$

(23) $$V_{\mathrm{lt}}=\sum q_{i}\left(C_{i}+{\mathrm{\alpha W}}_{i}\right)$$

(24) $$W_{i}=P_{i}∙r\left(T_{w}+T_{1}+T_{2}\right)$$

where *A*_g_ and *A* $V_{\mathrm{lt}}$ ($) measures the total tourist expenditure in the study area. $q_{i}$ (persons) quantifies the number of tourists from area *i*. $C_{i}$ ($) refers to the actual expenditure of consumers traveling (e.g., food, accommodation, transport, etc.). α denotes a parameter between 0 and 1. $\;W_{i}$ ($) indicates the expenditure of the time cost of traveling, and $P_{i}$ ($) stands for the wage rate in area *i*, which is the product of 1/365 and the total annual per capita wage in the *i* region. *r* (times) is interpreted as the number of trips. $T_{w}$, $T_{1}$, and $T_{2}$ (T) represent the tourists' time abandonment of working hours, transportation time, and playing time in the study area, respectively.

#### 2.2.9 Value Realization Rate of Ecosystem Products

Integrating existing knowledge regarding ecosystem products that can be practically utilized by humans (Zheng et al. 2023), we regard the value of grazing capacity and landscape tourism as representative of grassland ecosystem products that have already been transformed by the market. Based on this understanding, we formulated the equation to calculate the value realization rate of grassland ecosystem products as follows:

(25) $$R_{\text{GEPV}}=\frac{{\text{GEPV}}_{r}}{{\text{GEPV}}_{t}}$$

(26) $${\text{GEPV}}_{T}=V_{\mathrm{gc}}+V_{\mathrm{wr}}+V_{\mathrm{sr}}+V_{{\mathrm{co}}_{2}}+V_{\mathrm{op}}+V_{\mathrm{bio}}+V_{\mathrm{glt}}$$

where $R_{\text{GEPV}}$ (%) represents the value realization rate of grassland ecosystem products. ${\text{GEPV}}_{R}$ ($) is the value of grassland ecosystem products that have been realized through the market. Due to the public nature of the regulation service, its value is not realized through the market. The value realized in the three study areas is the value of grazing capacity and landscape tourism. ${\text{GEPV}}_{T}$ ($) measures the total value of grassland ecosystem products, which is the total value of grazing capacity ($V_{\mathrm{gc}}$), water retention ($V_{\mathrm{wr}}$), soil retention ($V_{\mathrm{sr}}$), carbon sequestration ($V_{{\mathrm{co}}_{2}}$), oxygen production ($V_{\mathrm{op}}$), biodiversity ($V_{\mathrm{bio}}$), and landscape tourism ($V_{\mathrm{lt}}$).

### 2.3 Analysis of Influencing Factors

First, we construct the indicator system of dependent and independent variables. The dependent variable includes GEP per unit area and the value realization rate of ecosystem products. The independent variable has 13 indicators (see Figure 1 for details). Second, we employed Pearson's correlation models to analyze the factors influencing the dependent variable. The correlation coefficients were determined through bivariate autocorrelation analysis using IBM SPSS Statistics 2022. The significance of the differences between variables was analyzed using one‐way ANOVA, while the significance of comparisons between multivariate variables was assessed using the least significant difference (LSD) method. Subsequently, we use the Random Forest model to measure and rank the relative importance of the influencing factors. As an integrated learning method based on decision trees, Random Forest provides prediction accuracy and stability by constructing multiple decision trees. Moreover, each decision tree is constructed based on random samples and random features, which can avoid the overfitting problem and has excellent robustness (Ramos et al. 2020). See Breiman (2001) for specific modeling principles and formulas. Finally, data visualization was performed using RStudio 4.4.1 and Origin 2024.

### 2.4 Data Sources

#### 2.4.1 Remote Sensing Data

**FIGURE 3:**
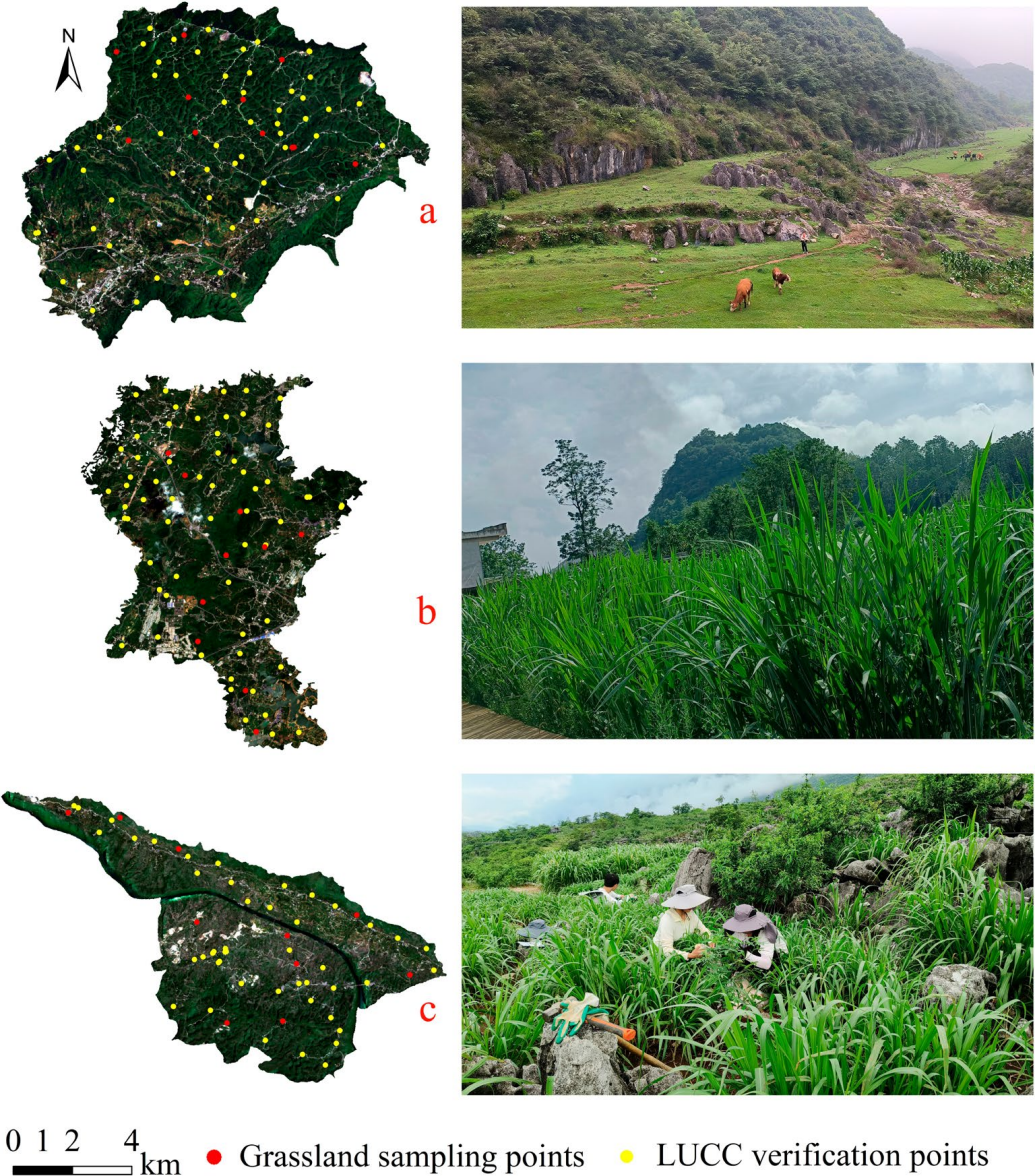
Land use validation sites, grassland sampling points, and photo verification in the study area. (a–c) represent the Salaxi, Hongfenghu, and Huajiang study areas, respectively. Photo by Yongyao Li.

1. LUCC: After comparing the advantages and disadvantages of Sentinel‐2, Landsat 9, and MODIS imagery (Table), we chose Sentinel‐2A remote sensing imagery as our data source. We retrieved and downloaded the data using July–October 2023 and cloud coverage of less than 5% as criteria. We conducted preprocessing steps, including atmospheric correction, radiometric calibration, image fusion and enhancement, geometric correction, image splicing, clipping, and mosaicing, to minimize various effects such as radiation and displacement. Referring to Bartold and Kluczek (2023) on Sentinel‐2A spectral, textural, and shape characterization, we used supervised classification, expert decision classification, and human–computer interactive interpretation based on the GEE platform for remote sensing interpretation and categorization of different land types. Building on this, a field survey and verification covering an area of approximately 1 × 1 km^2^ in the study region were conducted (Figure 3). Finally, we confirmed that the accuracy of the land use data exceeded 95%.

2 Bare rocky ratio: Referring to Zhao and Chen (2005) on the calculation method of the normalized difference bareness index, combined with the spectral characteristics of Sentinel‐2A remote sensing images, we calculate the normalized difference rock index (NDRI) by the following formula.

(27) $$\text{NDRI}=\frac{\text{Band}8-\text{Band}3}{\text{Band}8+\text{Band}3}$$

(28) $$F_{r}=\frac{\text{NDRI}-{\text{NDRI}}_{r}}{{\text{NDRI}}_{r}-{\text{NDRI}}_{0}}$$

where $F_{r}$ (%) refers to bare rocky ratio. NDRI is the normalized difference rock index. Band3 and Band8 represent the 3‐band and 8‐band of the image, respectively. $\;{\text{NDRI}}_{r}$ measures the pixel value of with rock outcrops, taken as the pixel value with a cumulative frequency of 99%. ${\text{NDRI}}_{0}$ represents the pixel value where no rock is exposed, and the pixel value corresponding to a cumulative frequency of 1% is utilized.

#### 2.4.2 Field Investigation Data

1. Plant diversity and fresh weight: According to the spatial distribution of natural and artificial grassland, we randomly arranged the sampling points according to the principle of the main distribution area of grassland and relative uniformity. The sampling points are shown in Figure 3. Due to the fragmented and dispersed nature of grasslands in the KDC area, coupled with the limited size of individual patches, we randomly set up three 1 × 1 m sample boxes within 100 m^2^ of grassland around each sample site. First, we identified the names of the herbaceous plants and classified the species types, and then we used Excel to record the number of individuals of each plant species according to the families and genera of the plants. We used the average of three sample sites to characterize a sample plot. The number of individuals of plants in each sample site is shown in Table S2. Subsequently, the entire above‐ground portion of the pasture was mowed to remove adhering soil, gravel, and other impurities, and the forage was weighed for fresh weight. At the same time, the plant root fresh weight was obtained by digging up the root system at the sampling site, washing it, and weighing it. See Table S2 for fresh weights of plants at sampling sites. Sampling was conducted in August 2023, the season when biomass is at its maximum.
2. Meteorological data: Data were collected from a small weather station in the study area (ATMOS, Meter Inc., USA). This weather station records meteorological data at a frequency of 30 min, ensuring the reliability of the data. The collected data were interpolated using the Kriging interpolation tool in ArcGIS.

#### 2.4.3 Laboratory Experimental Data

1. Dry weight of above‐ground and underground plants: The above‐ground plant material and underground roots were cut and excavated, then loaded into ice boxes and transported to the laboratory, where they were dried at 60°C and weighed. Plant dry weights at sampling sites are shown in Table S2.
2. Soil C, N, P, SOC, soil bulk density, and soil water content: Soil samples were collected in layers using a ring knife at the sampling points, with each layer defined as 0–10 cm, 10–20 cm, and 20–30 cm. After weighing the soil at the sampling site, the samples were transported to the laboratory using the ring knife for analysis of their physical properties. Soil moisture content was determined using the drying method, while soil bulk density and water holding capacity were assessed in the field using the ring knife method. Concurrently, soil samples from the same layer within each plot were thoroughly mixed to create composite samples for testing. These samples were then transported to the laboratory for natural air drying, debris removal, grinding through a 100‐mesh sieve, and subsequent bagging for storage. Finally, the chemical properties of the soil (organic matter, total nitrogen, total phosphorus, and total potassium) were determined. The experimental methods are detailed in Hu et al. (2022) and Liu et al. (2018).

#### 2.4.4 Collected Data

1. Demographic and socioeconomic data: There are nine, six, and six administrative villages in the Salaxi, Hongfenghu, and Huajiang study areas, respectively. We first went to the village committee of each village in the study area to collect population and labor data, including (10 indicators): the number of landscape tourists, landscape tourism income, distance to the county, distance to tourist attractions, total population, labor force population, number of migrant workers, number of high school graduates, number of pastoral practitioners, and per capita net income of farmers. On this basis, we conduct data correction through typical farmers to ensure the reliability of the data. We visited 211 farmers in the three study areas and obtained 211 questionnaires, 201 valid questionnaires with a weightage of 95.26%. Socioeconomic data for the administrative villages with the highest concentration of grasslands are shown in Table S5.
2. Terrain data (including elevation and slope): The data are from the National Aeronautics and Space Administration (https://www.nasa.gov), accessed on May 17, 2024.

## 3 Results

### 3.1 Supply Capacity of Grassland Ecosystem Products in the KDC Area

Significant differences were observed in the physical quantities of grassland ecosystem products per unit area across the different study areas. Overall, the Salaxi study area exhibited the highest values for water retention (36.69 mm/m^2^), soil retention (49.82 kg/m^2^), and biodiversity index (1.22) per unit area of grassland. This was followed by the Hongfenghu study area, which had values of 30.54 mm/m^2^, 13.85 kg/m^2^, and 0.88, respectively. The Huajiang study area recorded the lowest values, with 29.84 mm/m^2^, 3.52 kg/m^2^, and 0.4. These three indices exhibited a decreasing trend in physical quantities as the severity of karst desertification increased. In terms of grazing capacity, carbon sequestration, and oxygen production per unit area, Hongfenghu ranked first with 10.34 cattle/hm^2^, 748.53 t/hm^2^, and 512.51 g/m^2^, followed by Huajiang with 8.79 cattle/hm^2^, 579.25 t/hm^2^, and 380.96 g/m^2^. Salaxi ranked last with 6.94 cattle/hm^2^, 243.14 t/hm^2^, and 158.64 g/m^2^ (Table S3). This indicates a trend of light karst desertification accompanied by a decrease in physical quantities.

**FIGURE 4:**
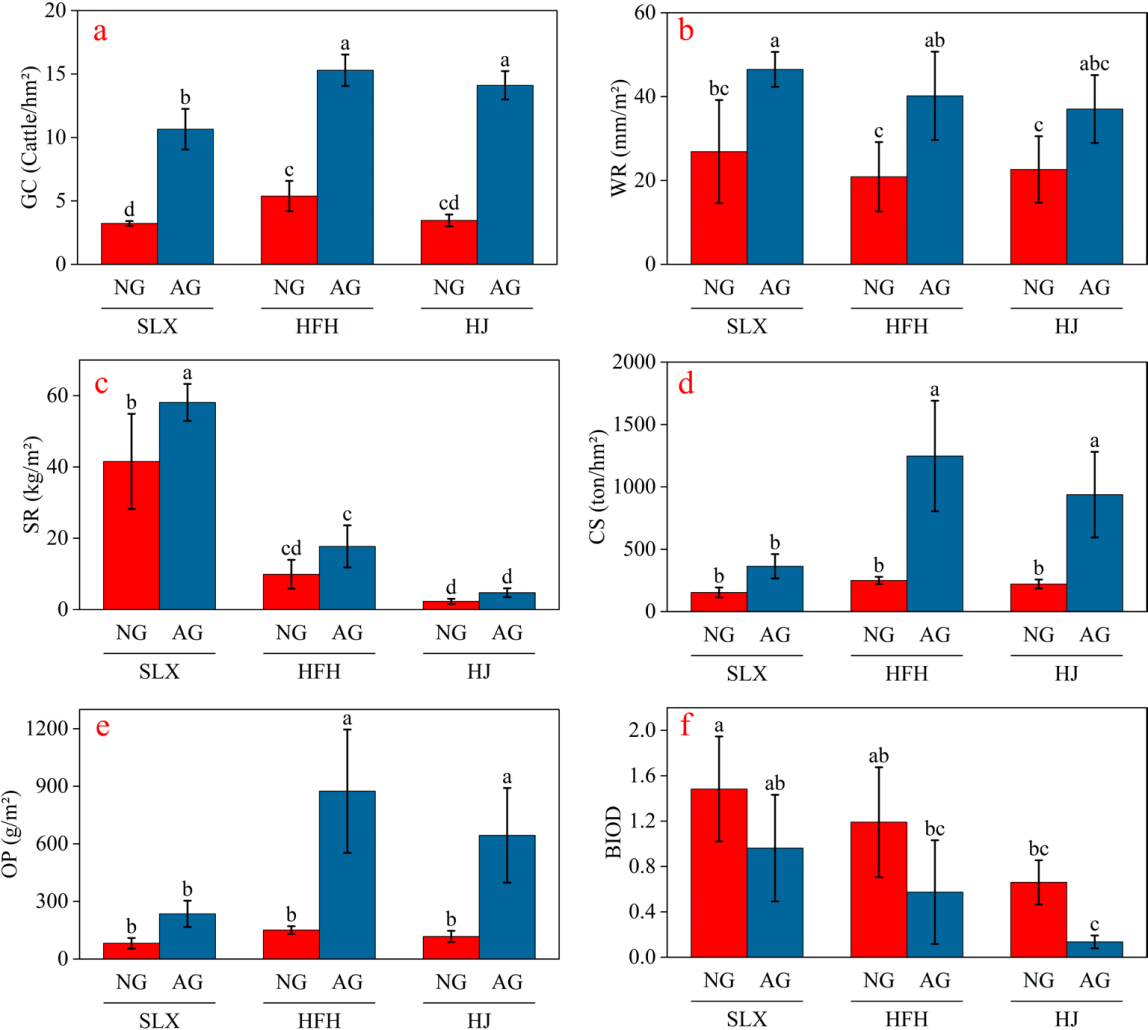
The physical quantity of grassland ecosystem products per unit area in the KDC area. Statistical significance level (*p* = 0.05) was evaluated using a one‐way analysis of variance with a one‐tailed test. AG, Artificial grassland; HFH, Hongfenghu; HJ, Huajiang; NG, Natural grassland; SLX, Salaxi..

The variability in the supply capacity of grassland ecosystem products among the three study areas is also reflected in the different grassland types. (1) Grazing capacity: significant differences were observed among the natural grasslands in the three study areas. In contrast, the artificial grasslands showed that Salaxi differed significantly from the other two study areas, while no significant difference was found between the artificial grasslands of Hongfenghu and Huajiang. Additionally, the grazing capacity of natural grasslands was lower than that of artificial grasslands, with a significant difference between the two (Figure 4a). (2) Water retention: the natural grassland in Salaxi exhibited significant differences from the other two study areas, while no significant difference was observed between Hongfenghu and Huajiang. Significant differences were also noted among the three study areas of artificial grassland. Furthermore, the water retention in natural grasslands was lower than that in artificial grasslands, with a significant difference between the two (Figure 4b). (3) Soil retention: a trend was observed with natural grassland exhibiting lower soil retention than artificial grassland. However, there was no significant difference between natural and artificial grasslands in Huajiang, while a significant difference was noted overall. Moreover, differences within the same grassland type were also pronounced (Figure 4c). (4) Carbon sequestration and oxygen release: No significant differences were found among the natural grasslands in the three study areas. In contrast, significant differences were observed between the artificial grasslands of Salaxi and those of Hongfenghu and Huajiang, while no significant difference existed between Hongfenghu and Huajiang (Figure 4d,e). (5) Plant diversity: The plant diversity in natural grasslands was higher than in artificial grasslands across all three study areas, with significant differences observed between the same grassland types. Notably, there was no significant difference between the artificial grassland in Salaxi and the natural grassland in Hongfenghu, nor between the artificial grassland in Hongfenghu and the natural grassland in Huajiang (Figure 4f).

### 3.2 Value Quantity and Value Realization Rate of Grassland Ecosystem Products in the KDC Area

#### 3.2.1 The Value of Each Individual Ecosystem Product

**FIGURE 5:**
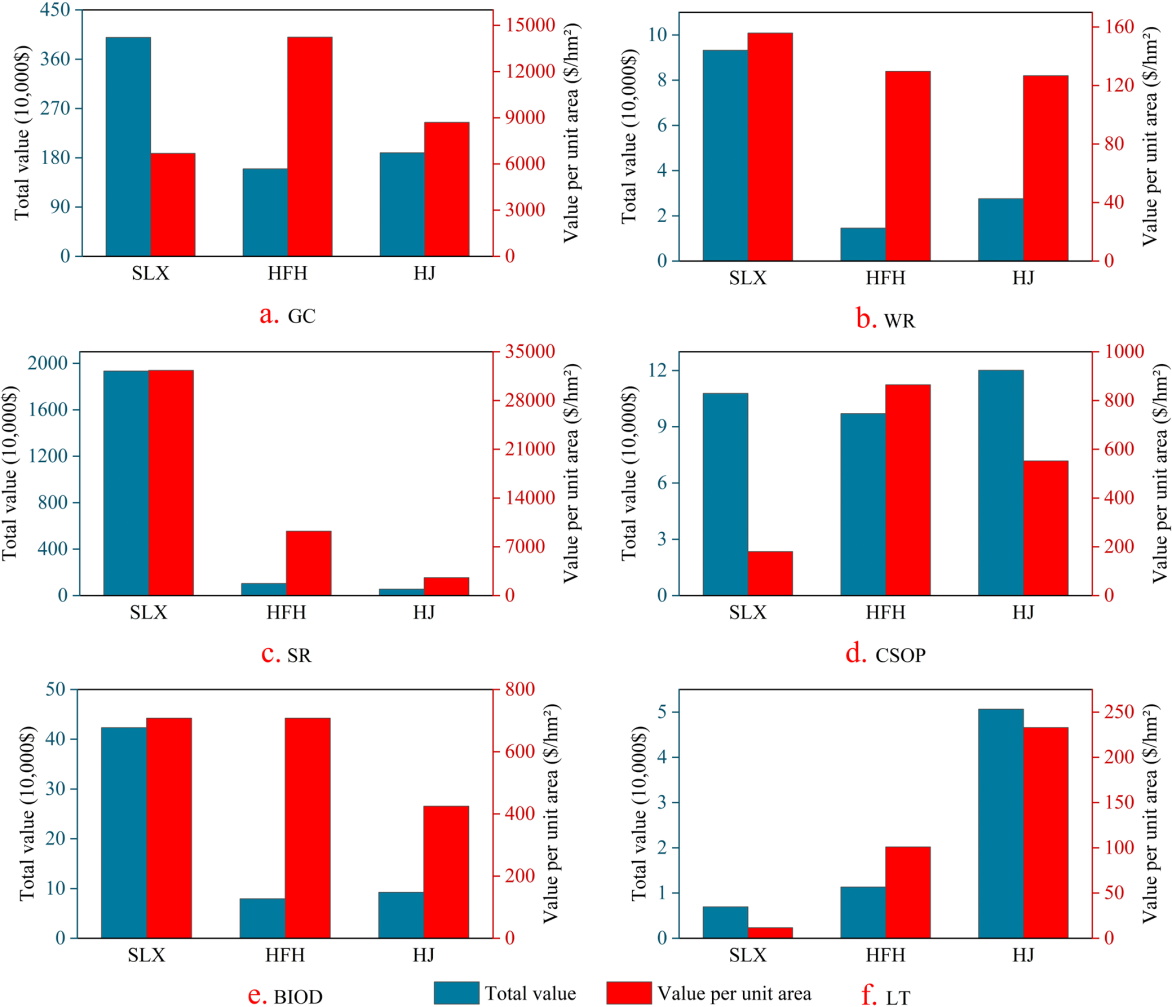
The total value of each individual ecosystem product and its value per unit area. (a–f) represent the total value and unit area value of grazing capacity, water retention, soil retention, carbon sequestration and oxygen production, plant diversity, and landscape tourism across the three study areas, respectively. BIOD, Biodiversity; CSOP, Carbon sequestration and oxygen production; GC Grazing capacity; HFH, Hongfenghu; HJ, Huajiang; LT, Landscape and tourism; SLX, Salaxi; SR, Soil retention; WR, Water retention.

As shown in Figure 5a–f and Table S4, the values of ecosystem products per unit area of grassland reveal that soil retention, grazing capacity, and biodiversity rank among the top three, while water retention, carbon sequestration and oxygen release, and landscape tourism occupy the bottom three positions. Regarding the value of grassland ecosystem products per unit area, the overall ranking is Salaxi > Hongfenghu > Huajiang, indicating that the value decreases with the increasing of karst desertification levels. Specifically, the average values for grazing capacity, as well as carbon sequestration and oxygen release, are ranked as Hongfenghu > Huajiang > Salaxi. Conversely, soil retention and water retention are ranked as Salaxi > Hongfenghu > Huajiang. The biodiversity value of grassland per unit area is ranked as Salaxi > Huajiang > Hongfenghu. The value of landform and tourism is ranked as Huajiang > Hongfenghu > Salaxi.

#### 3.2.2 GEP and Value Realization Rate

**FIGURE 6:**
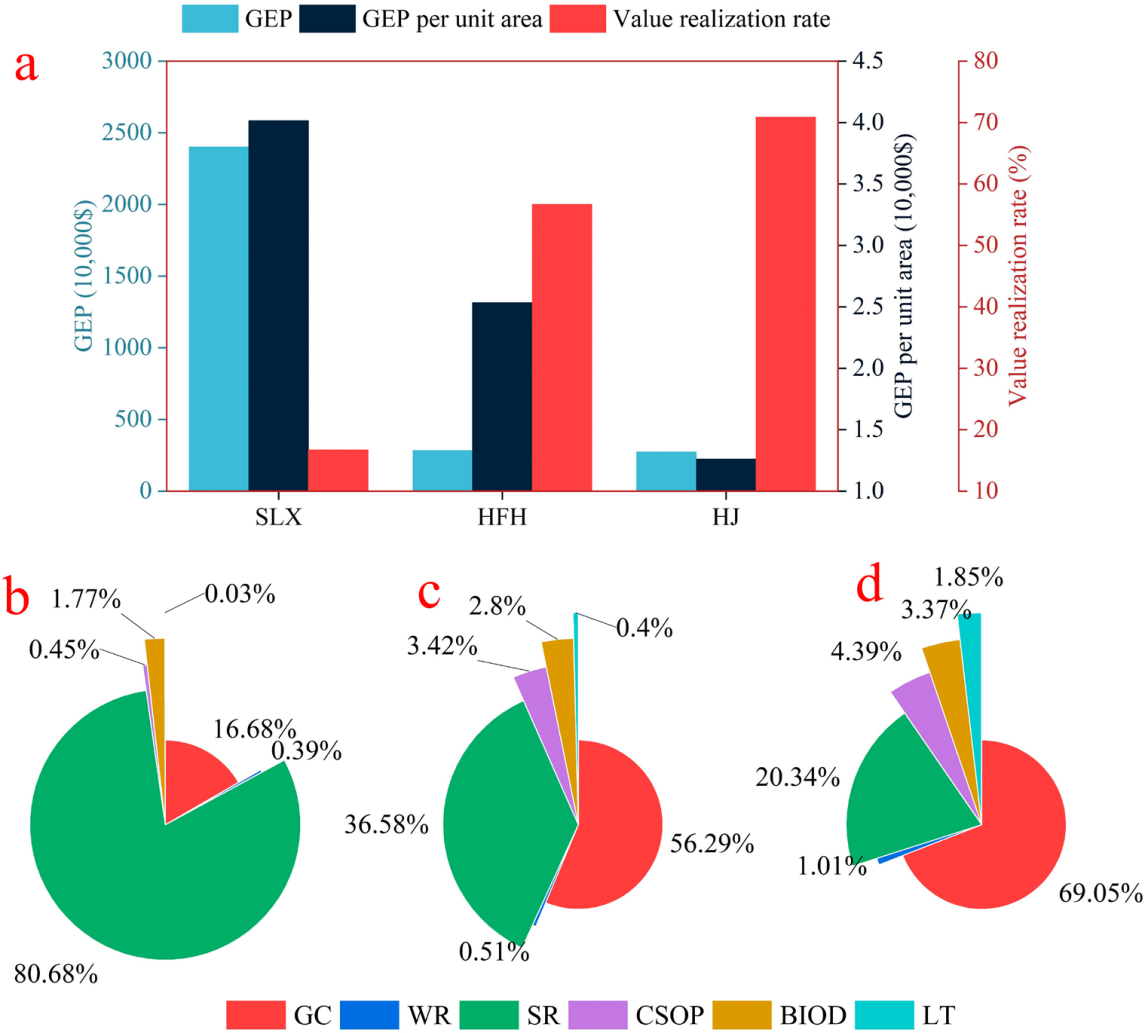
Distribution of grassland GEP and value realization rate in the KDC area. (a) represents the comparison of GEP, GEP per unit area, and value realization rates across the three study areas; (b) refers to the proportion of GEP structure in Salaxi; (c) describes the proportion of GEP structure in Hongfenghu; (d) illustrates the proportion of GEP structure in Huajiang. BIOD, Biodiversity; CSOP, Carbon sequestration and oxygen production; GC, Grazing capacity; GEP, Gross ecosystem products; HFH, Hongfenghu; HJ, Huajiang; LT, Landscape and tourism. SLX, Salaxi; SR, Soil retention; WR, Water retention.

As shown in Figure 6a, the GEP of grassland in the KDC area is ranked as follows: Salaxi ($24.02 million) > Hongfenghu ($2.84 million) > Huajiang ($2.75 million). The GEP per unit area exhibits a similar trend, with Salaxi ($40,160.64) > Hongfenghu ($25,345.20) > Huajiang ($12,630.03). This trend is primarily attributed to the structure of the GEP. In Salaxi, the values of soil retention, grazing capacity, and biodiversity are higher than those of other ecosystem products, which is why the GEP and GEP per unit area rank first (Figure 6b). Despite its smaller area, Hongfenghu demonstrates high productivity of grassland resources, with the GEP per unit area exceeding that of the Huajiang study area (Figure 6c). The Huajiang study area experiences a high degree of karst desertification and exhibits a low capacity for ecosystem product supply, resulting in its GEP per unit area ranking last (Figure 6d).

Notably, the realization rate of ecosystem product value for grasslands in the KDC area is ranked as follows: Huajiang (70.9%) > Hongfenghu (56.69%) > Salaxi (16.71%). This indicates that the value realization rate of grassland ecosystem products increases with the level of karst desertification. This phenomenon is primarily attributed to the higher proportions of grazing capacity value and landscape tourism value in the Huajiang study area compared to those in Hongfenghu and Salaxi, respectively (Figure 6a–d). Among the six types of grassland ecosystem products in the KDC area, only grazing capacity and landscape tourism values can be realized through market transactions. In contrast, the other four types of ecosystem products (water retention, soil retention, carbon sequestration and oxygen production, and biodiversity) cannot be monetized due to their externalities.

### 3.3 Drivers of GEP and Value Realization Rate of Grassland Ecosystem Products

#### 3.3.1 Intensity and Direction of the Influencing Factors

**FIGURE 7:**
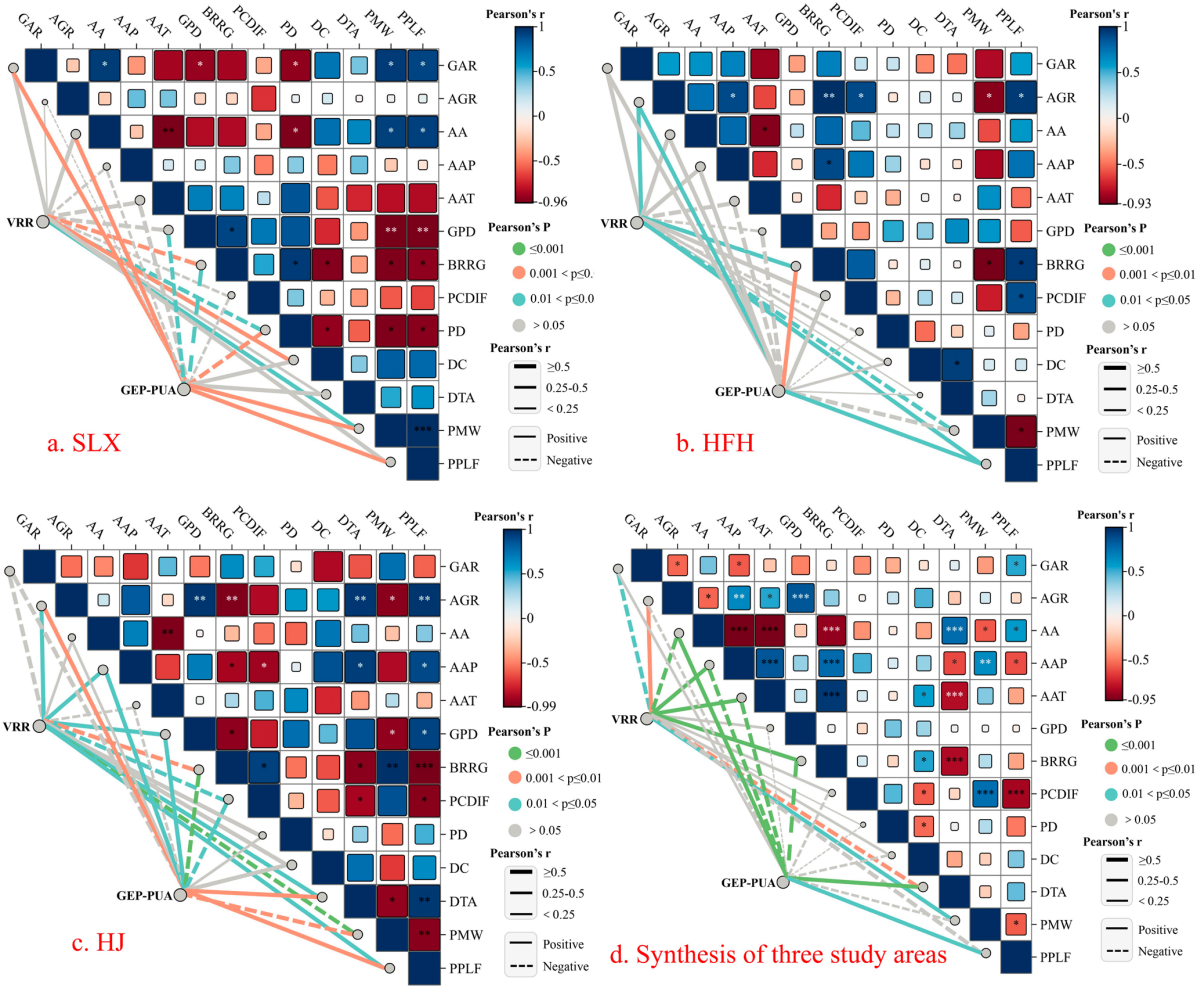
Factors influencing the GEP per unit area and the value realization rate of grassland ecosystem products in the KDC area. (a–d) represent Salaxi, Hongfenghu, Huajiang, and three study areas combined, respectively. AA, Average altitude; AAP, Average annual precipitation; AAT, Average annual temperature; AGR, Artificial grassland ratio; BRRG, Bare rocky ratio of grassland; DC, Distance to county; DTA, Distance to tourist attractions; GAR, Grassland area ratio; GEP‐PUA, GEP per unit area; GEP‐PUA, Gross ecosystem products per unit area; GPD, Grassland patch density; HFH, Hongfenghu; HJ, Huajiang; PCDIF, Per capita disposable income of farmers; PD, Population density; PMW, Proportion of migrant workers; PPLF, Proportion of pastoralists in the labor force; SLX, Salaxi; VRR, Value realization rate; VRR, Value realization rate.

As shown in Figure 7a, our bivariate correlation analysis revealed significant associations between GAR, AA, GPD, BRRG, PD, PWM, and PPLF and other indicators in Salaxi. This finding suggests that these indicators exert the most substantial influence. Regarding the influencing factors of GEP per unit area, GAR, AA, PWM, and PPLF have positive and significant effects; however, GPD, BRRG, and PD exhibit the opposite effect. This observation reflects that grassland ecosystem products are primarily distributed in areas with high grassland resource endowment, characterized by elevated altitudes, extensive grassland areas, and a significant population of migrant laborers, thereby facilitating greater engagement of farmers in pastoral production. Conversely, as grasslands become smaller and more fragmented, with increasing rates of rock exposure and higher population density, the production of grassland ecosystem products is inhibited. Regarding the value realization rate of ecosystem products, BRRG and PD exert negative and significant effects. This indicates that a higher rate of rock outcrop corresponds to lower grassland resource endowment, alongside an increased risk of intensive agricultural activities disturbing the grassland, thereby becoming a barrier to enhancing the value realization rate. DC and PMW demonstrate positive and significant effects, suggesting that greater distances from the county and a higher population of migrant workers are conducive to the development of the traditional cultivation industry toward animal husbandry.

In the Hongfenghu study area, significant correlations were observed between AGR, AA, AAP, BRRG, PCDIF, DC, PMW, PPLF, and other indicators. Factors affecting GEP per unit area of grassland in the KDC area were positively influenced by AGR, BRRG, and PPLF. Unlike in Salaxi, a higher rate of rock exposure in the Hongfenghu study area correlates with an increased capacity to supply ecosystem products. This correlation is closely associated with the presence of valuable local land resources and the practice of farmers planting artificial grasses in areas with high rock exposure. Regarding the factors influencing the value realization rate of ecosystem products, the study area is significantly and positively influenced by AGR, BRRG, and PPLF, while PMW exerts a significant negative influence. In contrast to Salaxi, a significant number of individuals from the Hongfenghu study area seeking employment elsewhere reduce the local labor force, thereby negatively impacting the value realization rate of ecosystem products. The development of local herbivorous livestock husbandry is characterized by intensive facility agriculture, marking a significant departure from the traditional grazing practices observed in Salaxi (Figure 7b).

In the Huajiang study area, a significant correlation exists between AGR, AA, AAP, GPD, BRRG, PCDIF, DTA, PMW, and PPLF, indicating a relatively high intensity of action. Notably, the significant factors affecting GEP per unit area and the value realization rate are consistent, with AGR, AAP, GPD, DTA, and PPLF exhibiting significant positive effects, while BRRG, PCDIF, and PWM demonstrate significant negative effects. In contrast to Salaxi, an increasing population of migrant laborers in the Huajiang study area negatively impacts the overall improvement of GEP. This phenomenon arises because the Huajiang study area requires a substantial number of laborers for cultivating *Pennisetum hydridum*, which is essential for developing animal husbandry. This observation aligns with the intensive agricultural development in the Hongfenghu study area; however, the industrial development in Huajiang remains relatively traditional and extensive. Nevertheless, the Huajiang study area differs from Hongfenghu regarding the rock exposure rate, as *Pennisetum hydridum* is primarily cultivated on land with relatively favorable soil conditions. Although the rock exposure rate in both Huajiang and Salaxi negatively impacts GEP, the two areas differ in that the high rock exposure rate in Salaxi is detrimental to grass growth and grazing. The Huajiang study area relies on the favorable land resources of neighboring farmers to establish artificial grasslands for animal husbandry development (Figure 7c).

When considering the three study areas collectively, the correlations among the indicators were significant, with the exception of GPD and DC, which lacked significant correlations with the other indicators. Regarding the factors influencing GEP per unit area, AA, DTA, and PPLF have significant positive effects, whereas AAP, AAT, and BRRG have significant negative effects. Concerning the value realization rate, GAR, AA, and DTA have significant negative effects, while AGR, AAP, AAT, BRRG, and PMW exhibit significant positive effects (Figure 7d). These findings suggest that, overall, socioeconomic development and human activities play a more significant role in influencing GEP per unit area and the value realization rate of grassland ecosystem products.

#### 3.3.2 Relative Importance of Influencing Factors

Based on the random forest model, the R^2^, mean absolute error (MAE), mean square error (MSE), root mean square error (RMSE), and mean absolute percentage error (MAPE) for the dependent variable GEP‐PUA, using 13 independent variables in the test set, were 0.632, 3.583, 13.886, 3.726, and 0.005, respectively. The R^2^, MAE, MSE, RMSE, and MAPE for VRR, using 13 independent variables in the test set, were 0.8, 6.025, 53.659, 7.325, and 0.007, respectively. The model demonstrates strong predictive performance for the dependent variables GEP‐PUA and VRR, with high explanatory power and accuracy. The high R^2^ value indicates that the model provides a strong fit to the data, while the low values of MAE, MSE, RMSE, and MAPE further validate its predictive accuracy.

**FIGURE 8:**
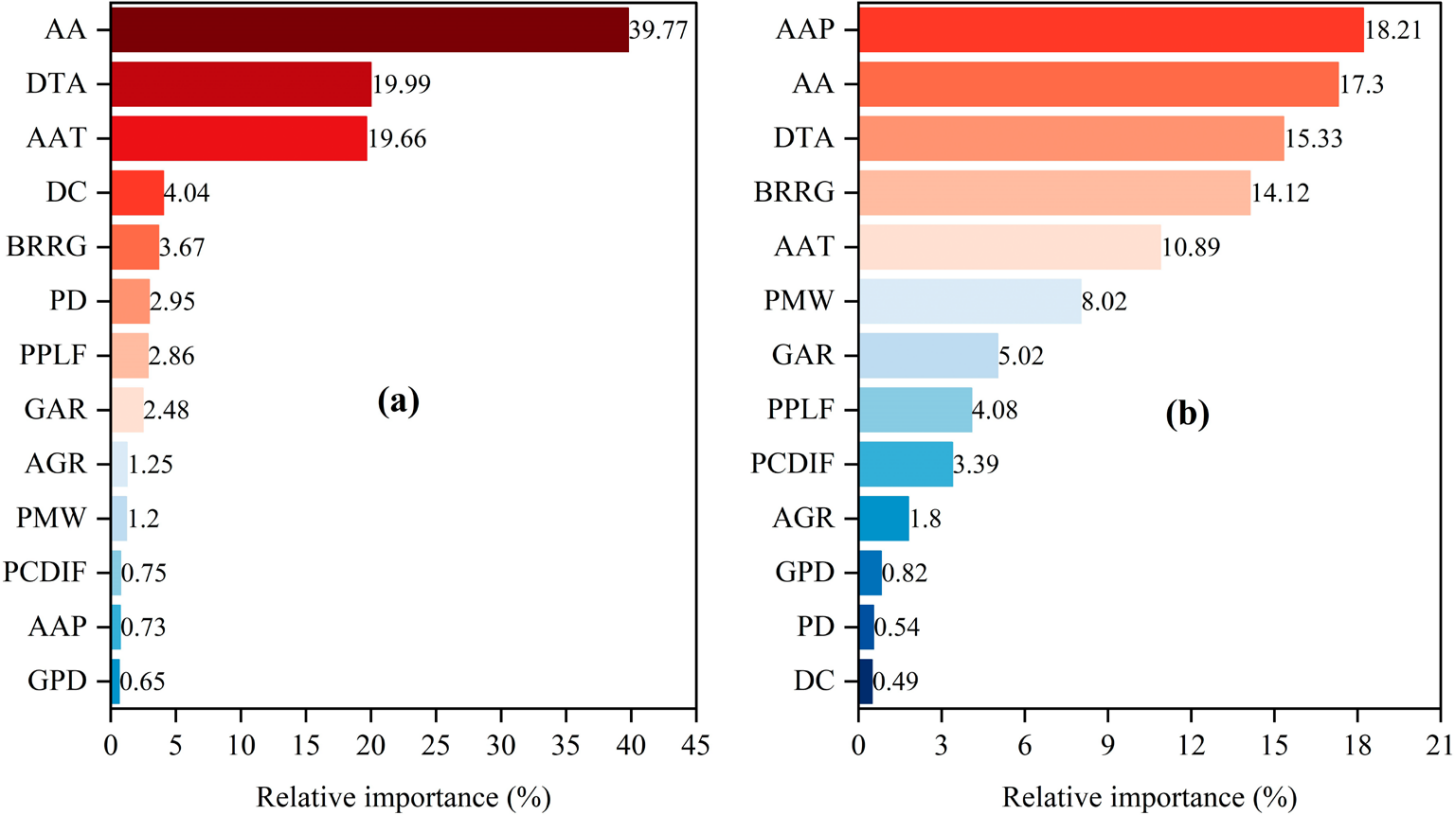
Relative importance of GEP‐PUA and VRR influences evaluated based on the random forest model. (a and b) represent the relative importance of GEP‐PUA and VRR influences, respectively. AA, Average altitude; AAP, Average annual precipitation; AAT, Average annual temperature; AGR, Artificial grassland ratio; BRRG, Bare rocky ratio of grassland; DC, Distance to county; DTA, Distance to tourist attractions; GAR, Grassland area ratio; GEP‐PUA, Gross ecosystem products per unit area; GPD, Grassland patch density; PCDIF, Per capita disposable income of farmers; PD, Population density; PMW, Proportion of migrant workers; PPLF, Proportion of pastoralists in the labor force; VRR, Value realization rate.

In terms of the relative importance of influencing factors, AA, DTA, and AAT were the most significant for GEP‐PUA, contributing 79.42% of the total weight, while the remaining 10 indicators accounted for 20.58% (Figure 7a). Regarding the influencing factors for VRR, five indicators showed relative importance greater than 10%, namely, AAP, AA, DTA, BRRG, and AAT, collectively accounting for 75.84% of the total weight. The combined weights of PMW and GAR were 13.04%, highlighting their notable contribution to the improvement of VRR. The remaining five indicators had a relatively small combined weight of 11.12% (Figure 7b). Overall, natural factors exhibit greater relative importance than human factors in influencing GEP‐PUA and VRR (Figure 8a‐b).

## 4 Discussion

### 4.1 Relationship Between Karst Desertification and the Grassland Ecosystem Products

Karst desertification resembles arid zone desertification and results from rock exposure due to vegetation destruction and soil erosion caused by human activities (Chen et al. 2021). The severity of karst desertification is closely related to rock exposure, vegetation cover, and soil cover (Zhang et al. 2021). Generally, a lower level of karst desertification corresponds to a thicker soil layer, a reduced rate of rock exposure, and more extensive vegetation development, leading to a higher supply capacity of grassland ecosystem products, and vice versa. This indicates that the supply capacity of grassland ecosystem products is constrained by the karst ecosystem. However, it is important to note that, in areas with high levels of karst desertification, higher temperatures and precipitation contribute to greater biomass in artificial grasslands compared to areas with lower karst desertification and less precipitation. As biomass increases, carbon sequestration and oxygen production also increase. This demonstrates that humans can modify the structure of ecosystem products through species breeding in areas with poor land resources but suitable climates to create artificial grasslands that better meet industrial needs.

Although our study demonstrated that areas with severe karst desertification can achieve high biomass for grass‐fed animal husbandry through appropriate species selection for artificial grasslands, establishing artificial grasslands is not a panacea. On one hand, the ability of artificial grassland establishment to sustain ecosystem health remains unproven. Previous studies have demonstrated that converting permanent grasslands into temporary grasslands diminishes ecosystem multifunctionality (Schils et al. 2022) and that land‐use intensification hinders biodiversity enhancement (Paudel et al. 2023). Our findings also indicate that artificial grasslands in areas with severe karst desertification exhibit low species diversity. Furthermore, artificial grasslands in the KDC region typically involve well‐defined stakeholder responsibilities and serve a relatively narrow group of beneficiaries, creating trade‐offs and equity challenges between ecosystem restoration and economic development. Consequently, the provision of grassland ecosystem products in the KDC region should be critically evaluated through the dual lenses of ecosystem health and human well‐being.

### 4.2 Drivers and Improvement Strategies of Grassland Ecosystem Products in the KDC Area

The supply capacity of grassland ecosystem products in the KDC region reflects a process where humans harness land productivity to obtain resources and support ecosystems, with herbaceous plants serving as a crucial intermediary. In this process, the elements of water, soil, atmosphere, biology, and rock within the grassland ecosystem interact to facilitate energy transformations. Rock fissures in karst desertification areas are extensively developed (Liu et al. 2019; Xiao and Xiong 2022) and exhibit poor water retention capacity. Plant water sources primarily depend on fissure water, leading to pronounced aridity in vegetation. Combined with widely exposed rocks (Deng et al. 2023; Xiong et al. 2023), microhabitat grasslands exhibit patchy, point‐like distributions. The imbalance of soil and water resources adversely impacts the productivity of grassland ecosystem products. Furthermore, human activities, including positive ecological engineering and negative disturbances, play a significant role. For instance, there is broad consensus that minimizing population disturbances and implementing ecological engineering enhances grassland ecosystem product production in karst areas with high spatial heterogeneity (Feng et al. 2024; Song et al. 2023). Conversely, the unsustainable development of grasslands on steep slopes exacerbates karst desertification.

It is widely acknowledged that the supply capacity of grassland ecosystem products is shaped by land resource endowment, climate, and human activities (Grime et al. 2000). However, variability in site conditions influences grasslands in the KDC region differently compared to other areas. In the KDC region, the supply of grassland ecosystem products is primarily constrained by abundant rocky outcrops and limited soil availability, whereas in other regions, climatic variability plays a more significant role. For instance, the productivity of alpine meadows on the Tibetan Plateau is constrained by low temperatures resulting from high altitude (Dong et al. 2020; Zhou et al. 2024). Temperate grasslands and savannas are primarily affected by drought (Wu et al. 2023; Sankaran and Staver 2019). Regarding human factors, the primary threat to grassland ecosystem products in KDC areas arises from land trade‐offs driven by the demand for productive, living, and ecological land (Li et al. 2023b; Li et al. 2024). In contrast, grassland degradation in other regions is primarily driven by unpredictable land‐use patterns, such as overgrazing (Ganguli and O'Rourke 2022; Erb et al. 2018), mineral exploitation (Qi et al. 2022), and excessive mowing (Bartold et al. 2024), all of which contribute to ecosystem destabilization. Nonetheless, all these issues are rooted in a significant human–land conflict. Therefore, it is essential to optimize the integration of soil and water resources and land use strategies, minimize anthropogenic disturbances to natural grasslands, and increase the proportion of artificial grasslands to comprehensively enhance the supply capacity of grassland ecosystem products in the KDC area.

### 4.3 Drivers and Recommendations of Value Realization Rate of Grassland Ecosystem Products in the KDC Area

Enhancing the value realization rate of ecosystem products is a universal challenge for all grasslands, including those in the KDC region. The value realization rate of ecosystem products depends not only on the total quantity of ecosystem goods and services but also on the relative proportion of individual ecosystem products. Generally, transforming ecosystem regulating services into economic value through market transactions is challenging due to market failures linked to their public nature (Bellanger et al. 2021). The larger the volume of competitive and exclusive ecosystem products, the greater the likelihood of their trade in the private market, resulting in a higher value realization rate. Grassland ecosystem products are shaped by trade‐offs and synergistic relationships (Hanisch et al. 2020), while these relationships, in turn, are influenced by habitat and plant functional traits (Mahaut et al. 2023). In contrast to the factors affecting the value realization rate of grassland ecosystem products in other regions, the grasslands in the KDC areas face constraints due to the fragile karst ecosystems. Considering these factors, it is evident that, given the specific land resource endowment and climatic context, artificial grassland can surpass the traditional limitations of ecological succession. By selecting and breeding species, it is possible to rapidly enhance biomass, thereby providing grass‐fed resources for pastoral development. Additionally, landscape tourism can increase the added value of grassland. The integrated development of grass‐fed animal husbandry and tourism can transform the structure of ecosystem products, thus enhancing their economic value.

In addition to the insufficiency of operational ecosystem products—those that can be traded in the market—low conversion rates of public ecosystem products to operational products also represent a significant and challenging factor affecting the value realization rate of grassland ecosystem products. While tools such as green finance (Wu et al. 2024), industrial restructuring (Zhu et al. 2024), and payment for ecosystem services (Dong et al. 2024) show potential for transforming ecosystems into market‐based economic benefits, their application in the KDC grasslands remains in its infancy and requires continuous practice. Moving forward, it is essential to develop a mechanism for internalizing the externalities of ecosystem products by integrating the interests and needs of stakeholders—such as governments, enterprises, social groups, and individual consumers—and fully leveraging the decisive role of the market.

### 4.4 Limitations and Next Step

Despite a considerable body of research on the value realization of ecosystem products, the concept continues to be a contentious topic in academic discourse. One perspective, for example, asserts that the essence of ecosystem product value realization lies in conceptualizing ecosystems as value creators, emphasizing the process of economic value transformation (Song and Du 2024). An alternative viewpoint suggests that the concept of ecosystem goods and services is not solely human‐centric; it also highlights the intrinsic value of both humans and their interdependent natural ecosystems (Costanza 2024). This paper adopts the former view, which overemphasizes the contribution of ecosystem material goods and cultural services to the economy while underappreciating the value of regulating services (e.g., biodiversity, soil retention, and water retention) to ecosystem health. Furthermore, it does not propose an economic‐ecological balance, which may be seen as controversial or biased.

Additionally, the spatial and temporal scales, as well as the indicator data utilized in this article, may have certain limitations. The study covers a 1‐year period; thus, the results may differ from those of future studies. For instance, rainfall influences net primary productivity (NPP), water conservation, and soil retention. Consequently, the results from a single year may be influenced by climate extremes (e.g., droughts or floods), which can affect the compositional structure of GEP. Additionally, since all three study areas are less than 100 km^2^ and the proportion of grassland within the national territory is relatively small, furthermore, the landscape pattern of grasslands is characterized by a mosaic distribution alongside other ecosystems, including forest ecosystems. Consequently, the physicochemical properties of plants and soil collected from grassland samples may be influenced by adjacent ecosystems. This may lead to an error range of the value of grassland ecosystem products, such as water conservation and soil preservation, when compared to isolated grasslands. Additionally, due to limitations in conditions, our biodiversity analysis considers only plant diversity, excluding animal and microbial diversity.

It is important to note that the factors influencing the grassland ecosystem products and their value realization rate are complex, with different scholars emphasizing different priorities. For example, Bartold and Kluczek (2024) emphasized the influence of grassland habitat and vegetation indices, Buzhdygan et al. (2020) explored the role of grassland biodiversity, and Richter et al. (2024) highlighted the driving role of grassland management. As research progresses, the indicator system, the influence of indicators, and their relative importance are likely to change.

In the future, researchers should aim to elucidate the “black box” of driving mechanisms underlying ecosystem product value realization by conducting grassland case studies across diverse temporal and spatial scales, grounded in a rigorous scientific definition of its scope and connotation. Simultaneously, based on local observations of the development of grassland ecosystem products, physical and numerical simulation studies should be encouraged to develop models suitable for the flow of grassland ecosystem products in the KDC area. These models should account for industrial development and the feedback loop between the economic system and the ecosystem. Specifically, it is essential to establish a measurement standard for balancing grassland ecology and the economy in the KDC area.

## 5 Conclusions

In this study, we quantified the physical quantity, value, and value realization rate of grassland ecosystem products across three different geomorphic types and levels of karst desertification, while analyzing the influencing factors. Our findings indicate that the physical quantity and value of grassland ecosystem products per unit area in the KDC area decreased as the severity of karst desertification increased; conversely, the value realization rate of ecosystem products increased. These findings substantiate the assertion that ecosystems have a significant capacity for product supply in regions with abundant grassland resources. However, they challenge the notion that the region can rapidly enhance the conversion rate of ecosystem products into market‐based economic value through industrial development. Our findings demonstrate that significant economic benefits can be achieved by enhancing grass yield in regions with highly fragile ecological environments through artificial grassland establishment. It is worth noting that the contribution of artificial grasslands should not be overstated, as their ecological sustainability remains contentious, evidenced by poor biodiversity, limited water conservation, and reduced soil retention capacities. Additionally, our findings indicate that the combined effects of water, soil, heat, and bare rock exert a greater influence on the supply capacity and value realization of grassland ecosystem products in the KDC area compared to human factors. Differentiated strategies for ecosystem protection, restoration, and industrial development should be implemented, taking into account the spatial variability of grassland terrain conditions in the KDC area. The results of this study offer valuable insights for transforming ecological advantages into economic benefits while maintaining ecosystem diversity, stability, and sustainability in ecologically fragile areas, particularly in the KDC area with similar conditions.

## Author Contributions

**Yongyao Li:** conceptualization (lead), data curation (lead), formal analysis (lead), investigation (lead), methodology (lead), resources (lead), software (lead), validation (lead), visualization (lead), writing – original draft (lead), writing – review and editing (lead). **Anjun Lan:** data curation (equal), formal analysis (equal), methodology (equal), software (equal). **Kangning Xiong:** funding acquisition (lead), supervision (lead). **Wenfang Zhang:** methodology (equal), resources (equal), software (equal), writing – original draft (equal). **Shuai Xiang:** methodology (equal), software (equal), visualization (equal). **Baoshan Zhang:** conceptualization (equal), investigation (equal), methodology (equal).

## Conflicts of Interest

The authors declare no conflicts of interest.

## Supporting information


**Table S1.** Advantages and disadvantages of Sentinel‐2A, Landsat 9, and MODIS remote sensing images.
**Table S2**. Survey results of grassland biomass and plant diversity in the karst desertification control area.
**Table S3**. Physical quantity of ecosystem products from natural and artificial grassland sampling sites in the three study areas.
**Table S4**. Data on value realization of grassland ecosystem products in the three study areas.
**Table S5**. Indicators of impact factors.

## Data Availability

Supporting Information (Table S1‐S5) associated with this article are available on Dryad: (https://doi.org/10.5061/dryad.6t1g1jx98).
