# Correction to “Value Realization of Grassland Ecosystem Products in the Karst Desertification Control Area: Spatial Variability, Drivers, and Decision‐Making”

**DOI:** 10.1002/ece3.71296

**Published:** 2025-04-21

Li Y, Lan A, Xiong K, Zhang W, Xiang S, and Zhang B. 2025. Value Realization of Grassland Ecosystem Products in the Karst Desertification Control Area: Spatial Variability, Drivers, and Decision‐Making. *Ecology and Evolution* 15: e71168. https://doi.org/10.1002/ece3.71168

There is a minor inconsistency between the funding information on the first page and the project number listed in the Acknowledgments section. To ensure accuracy, we have updated the Acknowledgments section to match the order and wording of the funding details provided on the first page. The correct acknowledgments statement should read as follows:

“This research was supported by the Philosophy and Social Science Planning Key Project of Guizhou Province (21GZZB43), the Key Science and Technology Program of Guizhou Province (6007 2014 QKHZDZXZ), and the China Overseas Expertise Introduction Program for Discipline Innovation (D17016).”

We apologize for this error.

